# Pharmacokinetics of Recombinant Human Soluble Thrombomodulin in Disseminated Intravascular Coagulation Patients with Acute Renal Dysfunction

**DOI:** 10.1160/TH16-07-0547

**Published:** 2017-02-23

**Authors:** Mineji Hayakawa, Shigeki Kushimoto, Eizo Watanabe, Koji Goto, Yasushi Suzuki, Toru Kotani, Takeyuki Kiguchi, Tomoaki Yatabe, Jun Tagawa, Fumiyo Komatsu, Satoshi Gando

**Affiliations:** 1 Emergency and Critical Care Center, Hokkaido University Hospital, Hokkaido, Japan; 2 Division of Emergency and Critical Care Medicine, Tohoku University Graduate School of Medicine, Tohoku, Japan; 3 Department of Emergency and Critical Care Medicine, Graduate School of Medicine, Chiba University, Chiba, Japan; 4 Department of Anesthesiology and Intensive Care Medicine, Faculty of Medicine, Oita University, Oita, Japan; 5 Department of Critical Care Medicine, Iwate Medical University, Iwate, Japan; 6 Department of Anesthesiology and Intensive Care Medicine, Tokyo Women’s Medical University, Tokyo, Japan; 7 Department of Emergency and Critical Care, Osaka General Medical Center, Osaka, Japan; 8 Department of Anesthesiology and Intensive Care Medicine, Kochi Medical School, Kochi, Japan; 9 Clinical Development Center, Asahi Kasei Pharma Co., Tokyo, Japan

**Keywords:** Plasma concentration, disseminated intravascular coagulation, pharmacokinetics, renal dysfunction, sepsis

## Abstract

Recombinant human soluble thrombomodulin (ART-123) is a novel anticoagulant for patients with disseminated intravascular coagulation (DIC). It is widely used in clinical settings throughout Japan. Furthermore, a global Phase 3 study is currently being conducted. In healthy subjects, ART-123 is excreted mainly via the kidneys. Therefore, ART-123 dose decrease was recommended in DIC patients with severe renal dysfunction. However, the pharmacokinetics of ART-123 in DIC patients with severe acute renal dysfunction has not been elucidated. In an open-label, multicentre, prospective, clinical pharmacological study, we investigated the pharmacokinetics and safety of ART-123 upon repeated administration to DIC patients. ART-123 was administered to patients at a dose of 130 or 380 U/kg/day for six consecutive days. Plasma concentrations of ART-123 were measured at 21 time points until eight days after the final administration. Urinary excretion rates during the first 24 hours (h) were calculated. Patient renal functions were evaluated by measuring 24-h creatinine clearance (Ccr). Forty-three patients were enrolled in the present study. The urinary excretion rates of ART-123 correlated closely with 24-h Ccr. Total body clearance of ART-123 was also weakly related with 24-h Ccr. However, the plasma concentrations of ART-123 were not considerably different among patients with different renal function. Two patients had subcutaneous haemorrhage as an adverse event related to ART-123. In conclusion, plasma concentrations of ART-123 may not be different among patients with different renal functions. ART-123 was well tolerated in these patients.

## Introduction

Thrombomodulin is a thrombin and protein C receptor on endothelial cell surface and plays an important role in the regulation of coagulation and the innate immune system (1). It has high affinity for thrombin and directly inhibits thrombin activity by forming a thrombin-thrombomodulin complex, which activates protein C and inhibits activated factor V and factor VIII (2–6). Although protein C is also activated by thrombin, the activation of protein C is amplified >1,000-fold by the thrombin-thrombomodulin complex (7). Furthermore, the activation of protein C by the thrombin-thrombomodulin complex is enhanced >20-fold when protein C is bound to the endothelial cell protein C receptor (8). Recombinant human soluble thrombomodulin (ART-123, Asahi Kasei Pharma Co., Tokyo, Japan), a novel anticoagulant, was approved in Japan in 2008 for patients with disseminated intravascular coagulation (DIC) (2–4). It has an active extracellular domain, can bind to thrombin, and can activate protein C similar to thrombomodulin (2). ART-123 was shown to have a unique mechanism of action, in which thrombin generation was suppressed via activation of protein C without direct inhibition of thrombin activity at therapeutic plasma concentrations (2). A global Phase 2 study showed the safety and efficacy of ART-123 in patients with sepsis and DIC (5). Based on this, a global Phase 3 study is currently ongoing in patients with severe sepsis and coagulopathy (6).

Recently, the clinical effects of ART-123 in patients with sepsis-induced DIC were reported (9–14). In our multicentre, retrospective, large-scale study, 45 % of sepsis-induced DIC patients treated with ART-123 had undergone renal replacement therapy (12). Rangel-Frausto et al. (15) reported that the percentage of sepsis patients with acute renal failure correlated with sepsis severity as well as with DIC complications. Additionally, in recent clinical settings, DIC was reported as an independent predictor of organ dysfunction, including acute renal dysfunction (16–18). Therefore, renal function should be considered when treating sepsis patients with renally excreted drugs.

As reported in a Phase 1 study, ART-123 is excreted mainly via the kidneys (19, 20). Therefore, ART-123 dose decrease was recommended in DIC patients with severe renal dysfunction. However, the pharmacokinetics of ART-123 in DIC patients with severe renal dysfunction have not been sufficiently elucidated thus far. We previously reported that the pharmacokinetic parameters of ART-123 in DIC patients with severe renal dysfunction were only slightly different from those in DIC patients without severe renal dysfunction (21). However, previous studies were based on single administration. Furthermore, based on pharmacokinetic simulations, we predicted that the plasma concentrations of ART-123 would not be different between DIC patients with and without severe renal dysfunction after repeated administration of a normal ART-123 dose (380 U/kg) (21).

To confirm the pharmacokinetic simulation results obtained in the previous study (21), we planned a prospective, multicentre, observational study for evaluating the pharmacokinetics of ART-123. DIC patients were classified into four groups based on 24-hour (h) creatinine clearance (Ccr). The pharmacokinetics and safety of ART-123 were investigated in these patients.

## Materials and methods

This study was conducted in patients who had been diagnosed as having DIC according to the Japanese Association for Acute Medicine (JAAM) DIC diagnostic criteria (16, 22). Other inclusion criteria were hospitalised patients aged at least 20 years. The main exclusion criteria were a high risk for fatal or life-threatening bleeding events; intracranial, gastrointestinal, or pulmonary haemorrhage; history of hypersensitivity to ART-123; pregnancy, breastfeeding, or possibly pregnancy; intermittent haemodialysis for a disease such as chronic renal failure; concomitant fulminant hepatitis, decompensated liver cirrhosis, aspartate transaminase ≥ 500 IU/l, alanine transaminase ≥ 500 IU/l or total bilirubin ≥ 10 mg/dl; or administration of thrombomodulin alpha (recombinant) (Recomodulin^®^ Injection 12800) within 30 days before the start of ART-123.

This study was conducted according to Good Clinical Practice (GCP), Good Post-marketing Study Practice (GPSP), and the Declaration of Helsinki. Prior approval was obtained from the Institutional Review Board of all participating institutions. Written informed consent was obtained from all patients or acceptable representatives.

This was an open-label, multicentre, clinical pharmacological study. Patients whose eligibility was confirmed by the principal investigator or co-investigator were enrolled in the study. ART-123 was administered at a dose of 380 U/kg/day (0.06 mg/kg/day) for six days, except for when the investigators judged that dose reduction (130 U/kg/day [0.02 mg/kg/day]) was necessary. ART-123 was administered to patients at a dose of 380 U/kg/day (or 130 U/kg/day), once daily, via drip infusion over a period of 30 minutes (min) for six consecutive days. The starting day of administration of ART-123 was defined as Day 1. The pharmacokinetics of ART-123 was examined every day of administration (Day 1; pre-dose, immediately after dosing, and 2, 4, and 8 h after dosing, Day 2-Day 5; pre-dose, immediately post-dose. Day 6; pre-dose, immediately post-dose, 2, 4, and 8 h), and 1 day, 2 days, and 8 days after the last administration. Urinary excretion rates were calculated in the 24-h period from dosing on Day 1 to dosing on Day 2. Adverse events, which were defined as any undesirable experiences regardless of the causal relationship to the study drug, were recorded during the period from dosing on Day 1 to 8 days after the last administration.

Prohibited concomitant medications included drugs under development, thrombomodulin alpha, and thrombolytic agents. Other drugs, including anticoagulant agents and antiplatelet agents, were allowed to be used concomitantly. Patients were also prohibited from receiving intermittent haemodialysis during the period from consent until the collection of blood and urine samples scheduled on Day 2 before the administration of study drug.

Patient conditions were evaluated based on the Acute Physiology and Chronic Health Evaluation (APACHE) II score. APACHE II scores were calculated using the data collected on the day before the start of study drug administration. The Sequential Organ Failure Assessment (SOFA) score and the JAAM DIC diagnostic criteria score were calculated using the data recorded before study drug administration on Day 1. The renal function of patients was classified based on 24-h Ccr, which was calculated using the 24-h (from dosing on Day 1 to 24 h after the administration) urine and serum creatinine data. The renal function of patients was classified into the following four groups: Ccr < 10 ml/min (Group 1), Ccr ≥ 10 ml/min and < 30 ml/min (Group 2), Ccr ≥ 30 ml/min and < 60 ml/min (Group 3), and Ccr ≥ 60 ml/min (Group 4).

Samples for the measurement of plasma ART-123 concentrations were collected in sodium heparin vacuum blood collection tubes and cryopreserved at –80 °C until measurement. The plasma and urine concentrations were measured using a validated enzyme-linked immunosorbent assay (ELISA) method using two types of mouse monoclonal antibody. The quantification limits of ART-123 were 5 ng/ml in plasma and 0.4 ng/ml in urine. This ELISA system was validated and showed good inter-assay accuracy (2.2 to 10.7 %) and precision (0.7 to 6.4 %). The principles and methods of the ART-123 quantification assay in urine were identical to those of the plasma assay. The difference in the quantification limits of the urine and plasma assays could be attributed to the different compositions of the samples (urine or plasma) used.

Indicators of pharmacokinetic parameters used in this study are as follows: CL is the volume of plasma that is totally cleared of its drug content per unit time and body weight (23). Vd represents the apparent volume into which a drug distributes (23). Total body clearance (CLtot) was calculated using the following equation:

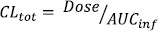

where AUC_inf_ is the area under the plasma ART-123 concentration-time curve from time 0 to time infinity.

Fractional excretion in urine from time 0 to 24 h after dosing (fe_0–24_) was calculated using the following equation:

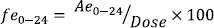

where Ae_0–24_ is the amount of ART-123 excreted in urine from time 0 to 24 h after dosing.

ART-123 pharmacokinetic parameters for the first 24 h were calculated by a non-compartmental model using WinNonlin software (version 6.3; Pharsight, Mountain View, CA, USA). SAS 9.2 software (SAS Institute Japan) was used for statistical analysis of the pharmacokinetic and safety data. The target sample size was set for each level of renal function at 5, 15, 10, and 10 patients for Group 1, Group 2, Group 3, and Group 4, respectively, to recruit a total of 40 patients.

## Results

### Disposition and demographics of patients

Of the 45 patients who gave consent during the period from November 2012 to June 2014 at eight investigational sites in Japan, 43 patients were enrolled in the study and received the study drug. Two patients met the exclusion criteria and were not enrolled. The safety analysis set included all 43 patients who received the study drug. The pharmacokinetic analysis set included 39 patients excluding four patients whose predefined dose could not be administered owing to errors in recording body weight or administration.

Patient demographics are presented in ►Table 1. The patients in Group 1 showed a trend toward higher APACHE II and SOFA scores than did the patients in other groups, because of severe renal dysfunction. In addition, more patients in Group 1 were receiving continuous renal replacement therapy (CRRT) as compared to the patients in other groups. Furthermore, regardless of the level of renal function, infectious diseases accounted for the majority of underlying diseases that directly induced DIC (►Table 1).

### Pharmacokinetics

#### Pharmacokinetics on Day 1

**Figure 1:**
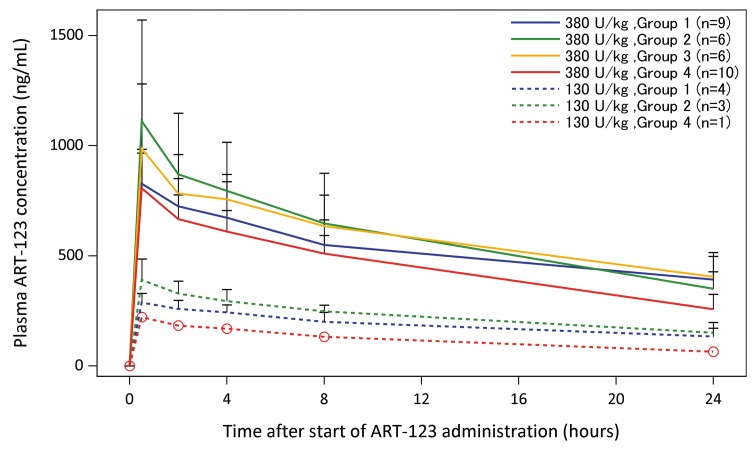
**Renal function effect on ART-123 plasma concentrations during the first 24 hours.** The plasma concentrations of ART-123 are presented as mean ± standard deviation. No relevant differences in the ART-123 plasma concentrations were observed among patients with different levels of renal function. Regardless of patient renal function, the plasma concentrations of ART-123 in disseminated intravascular coagulation (DIC) patients administered 380 U/kg of ART-123 were higher than those in the DIC patients administered 130 U/kg of ART-123.

The plasma concentrations of ART-123 during the first 24 hours are presented in ► Figure 1. The plasma ART-123 concentration in patients receiving either 380 U/kg or 130 U/kg peaked (C_max_) immediately after the completion of drug administration, and gradually decreased until drug administration on Day 2 (24 h after start of the drug administration on Day 1). No differences were observed in ART-123 plasma concentrations among patients with varying levels of renal function. Regardless of patient renal function, ART-123 plasma concentrations in DIC patients administered 380 U/kg of ART-123 were higher than that in DIC patients administered 130 U/kg of ART-123. The pharmacokinetic parameters of ART-123 during the first 24 h are presented in ► Table 2. Of the patients administered 380 U/kg of ART-123, patients in Group 1, 2, and 3 had reduced clearance, prolonged t_1/2_, and increased AUC_0–24_, compared to that reported for Group 4 patients.

**Figure 2:**
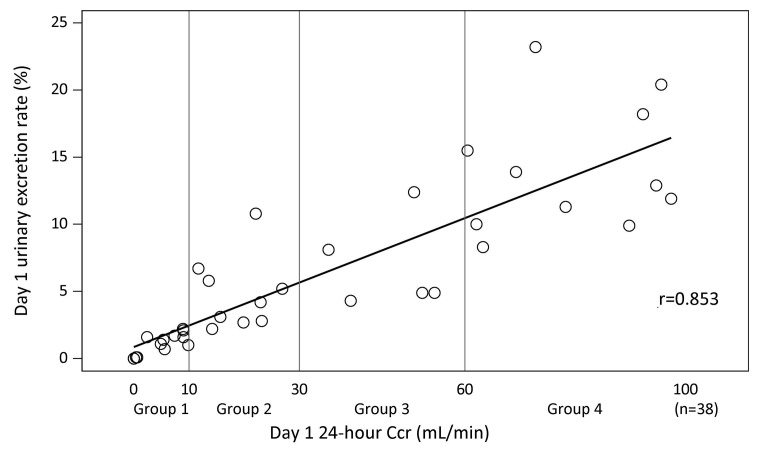
**Relationship between 24-h creatinine clearance and urinary excretion rate of ART-123 on Day 1.** The urinary excretion rate of ART-123 correlated closely with 24-h creatinine clearance. Urinary excretion rate of ART-123 was calculated by dividing the amount of ART-123 in urine excreted during 24 h after dosing by the amount of initially administered dose.

**Figure 3:**
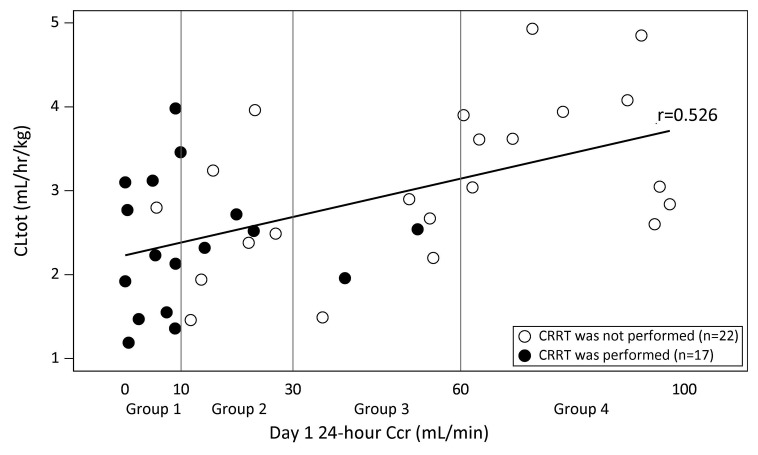
**Relationship between 24-h creatinine clearance and total body clearance of ART-123 on Day 1.** Total body clearance decreased in patients with decreased creatinine clearance (r = 0.526). However, the total body clearance in patients with creatinine clearance < 30 ml/min did not decrease. Although some patients received continuous renal replacement therapy (CRRT), it did not affect ART-123 clearance. ○ : CRRT was not performed, • : CRRT was performed. CLtot, total body clearance.

The relationship between the urinary excretion rate of ART-123 and 24-h Ccr is presented in ► Figure 2. The urinary excretion rate of ART-123 correlated closely with Ccr (r = 0.853). ► Figure 3 shows a scatter diagram of the 24-h Ccr and ART-123 total body clearance on Day 1. The total body clearance was weakly related to the 24-h Ccr (r = 0.526). However, the total body clearance in patients with Ccr < 30 ml/min did not decrease. Although some patients received CRRT, it did not affect their ART-123 clearance.

### Changes in ART-123 plasma concentration during six days repeated administration

**Figure 4:**
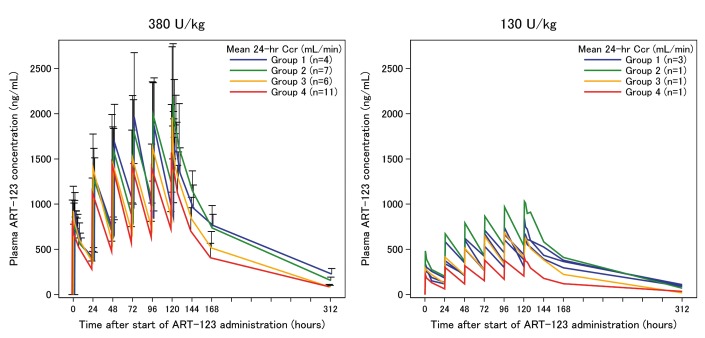
**Changes in ART-123 plasma concentration during six days repeated administration.** The left side shows the changes in ART-123 plasma concentration in patients administered 380 U/kg ART-123. The right side shows the changes in ART-123 plasma concentration in patients administered 130 U/kg ART-123. ART-123 plasma concentrations were stratified based on the mean 24-h creatinine clearance over six days. During the six-day observation period, 24-h creatinine clearance was measured daily. Among the patients administered 380 U/kg ART-123, one patient was excluded from the analysis of the ART-123 plasma concentrations due to lack of data for 24-h creatinine clearance on Day 6. No relevant differences in the ART-123 plasma concentrations were observed among patients with different levels of renal function. Furthermore, regardless of renal function, ART-123 plasma concentrations in patients administered 380 U/kg of ART-123 were higher than those in patients administered 130 U/kg of ART-123.

Among the 43 patients enrolled in the present study, 35 patients completed the repeated administration of ART-123 six times for six days (once daily). The changes in ART-123 plasma concentration during repeated administration for six days are presented in ► Figure 4. No relevant differences in the ART-123 plasma concentrations were observed among patients with different levels of renal function. Furthermore, regardless of renal function, ART-123 plasma concentrations in patients administered 380 U/kg of ART-123 were higher than those in patients administered 130 U/kg of ART-123.

### Safety

**Figure 5:**
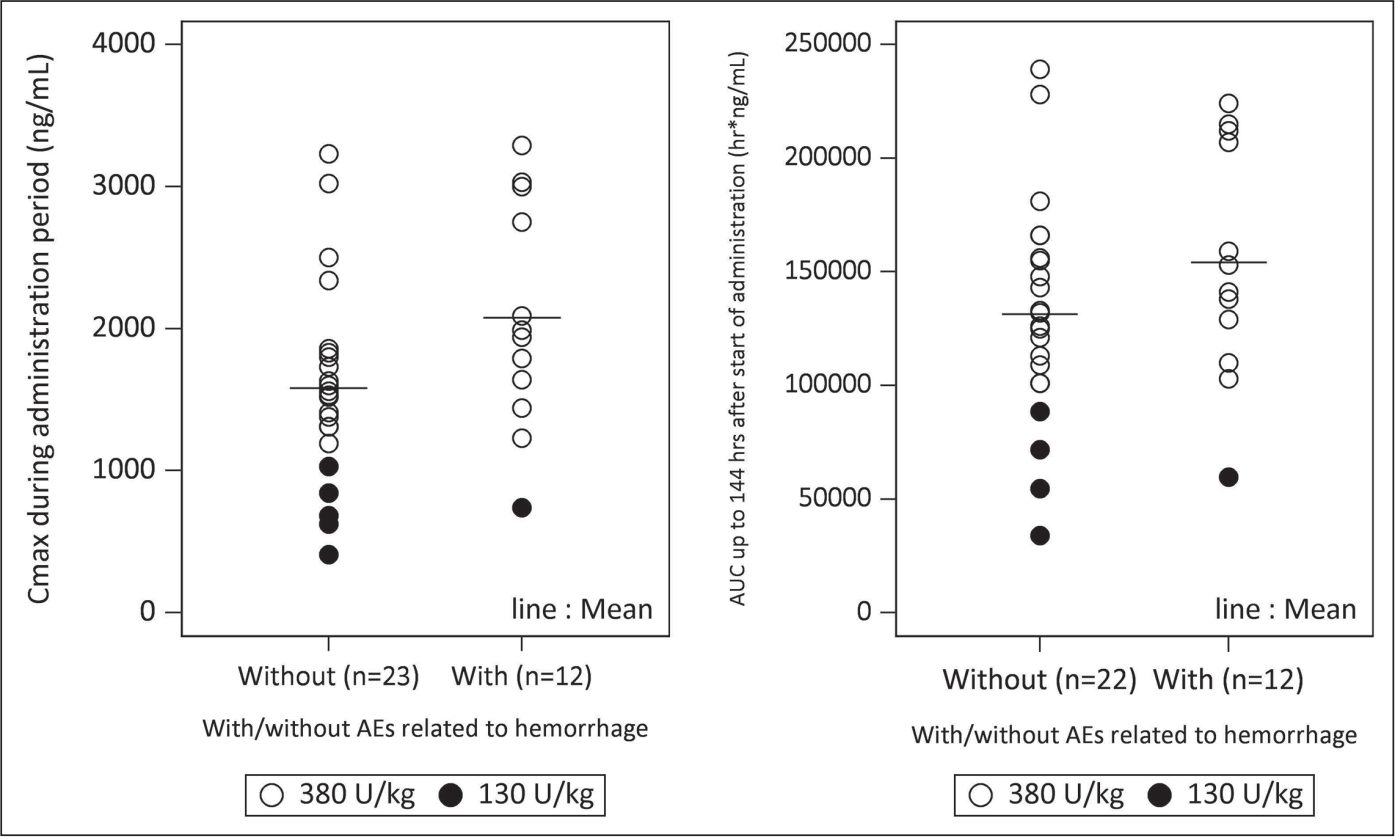
**Relationship between haemorrhagic adverse events and pharmacokinetic parameters.** Maximum plasma concentration (C_max_) or area under the plasma concentration-time curve during the first 144 h (6 days) (AUC_0–144_) in patients with and without haemorrhagic adverse events was not different. ○ : 380 U/kg, • : 130 U/kg. AEs, adverse events, defined as any undesirable experience regardless of the causal relationship to the study drug.

Haemorrhagic adverse events were observed in 39.5 % (17/43) of the patients enrolled in the present study. Of these, haemorrhagic adverse events related to ART-123 were observed in only two patients. Both cases were of subcutaneous haemorrhage. Furthermore, drug eruption (adverse event related to ART-123) was observed in one patient. Although one patient had anaemia induced by subcutaneous haemorrhage (a serious adverse event related to ART-123), the patient recovered eight days after onset. The distribution of C_max_ or area under the plasma concentration-time curve during the first 144 h (6 days) (AUC_0–144_) in patients with and without haemorrhagic adverse events is shown in ► Figure 5. Although C_max_ and AUC_0–144_ were not different between patients with and without haemorrhagic adverse events, correlations between haemorrhagic adverse events and C_max_, and between haemorrhagic adverse events and AUC_0–144_ cannot be ruled out.

## Discussion

We investigated the pharmacokinetics of ART-123 in four groups of DIC patients with renal dysfunction classified by 24-h Ccr. It was found that the urine excretion rate of ART-123 in these patients closely correlated with renal function. Although the total body clearance was weakly related with 24-h Ccr, the plasma concentration of ART-123 was not affected by renal dysfunction.

ART-123 is excreted mainly via the kidneys (19, 20, 23). In healthy subjects, 40 % of administered ART-123 was excreted into the urine during the first 24 h after administration (19, 20). Another study showed that administered ART-123 was excreted in the unchanged form (40 %) or metabolised form (55 %) into the urine during the first 24 h in normal control rats (23). Two previous population pharmacokinetic analyses using data from healthy subjects and DIC patients showed that renal dysfunction only slightly affected the pharmacokinetics of ART-123 (24, 25). Therefore, plasma concentrations of ART-123 were not significantly affected by renal function in those studies. However, these analyses did not correctly evaluate renal function because they used the Cockcroft-Gault equation (24, 25). In the present study, we evaluated renal function using 24-h Ccr and the urinary excretion rate of ART-123. The urinary excretion rate of ART-123 during the first 24 h correlated closely with the 24-h Ccr. However, even in DIC patients with normal renal function, less than 20 % of the administered ART-123 was detected in urine (► Table 2). Furthermore, the total body clearance of ART-123 was weakly related with 24-h Ccr, similar to previous population pharmacokinetic analyses (24, 25). The plasma concentrations of ART-123 in patients with and without severe renal dysfunction were not significantly different during repeated administration of ART-123. In DIC patients, other metabolic processes, such as degradation by the activated elastase, may contribute to ART-123 pharmacokinetics (26, 27). Therefore, the effects of renal excretion on total clearance of ART-123 may be relatively reduced in DIC patients.

Until now, there were no reported studies related to the organs or mechanisms involved in the metabolism of ART-123. However, it was believed that a scavenger-receptor-mediated non-specific or specific uptake contributes to the metabolism of high-molecular-weight protein drugs such as ART-123 (28, 29). Tsuruta et al. showed that the concentration of unmetabolised ART-123 in the liver, kidney, and lung was lower than that in the plasma of rats (23). They suggested that there were no specific organs in which ART-123 was distributed at concentrations higher than that in the plasma. Therefore, it is likely that scavenger-receptor-mediated non-specific uptake or metabolism is involved in the metabolism of ART-123, suggesting that ART-123 is metabolised in various organs.

Although the additional elimination enforced by CRRT generally affects the pharmacokinetics of low-molecular-weight drugs (such as antibiotics), the pharmacokinetics of high-molecular-weight proteins, such as albumin (56,000 Da), was reported to be unaffected (30, 31). A similar trend was observed for the pharmacokinetics of ART-123 (62,000 Da) (32). In the present study, CRRT was performed in 14 (35 %) patients with renal dysfunction. However, it seemed to have no impact on the pharmacokinetics of ART-123, which was in agreement with a previous study (21).

A Phase III study of ART-123 in Japan showed that the number of haemorrhagic adverse events associated with ART-123 in the DIC patients was markedly lower than that associated with heparin (2). In the present study, some haemorrhagic adverse events were observed. Although C_max_ and AUC_0–144_ were not different between patients with and without haemorrhage, correlations between haemorrhagic adverse events and both C_max_ and AUC_0–144_ could not clearly be ruled out. The plasma concentration of ART-123 required for direct inhibition of thrombin activity (8,200 ng/ml, concentration needed to double thrombin time) was about 200 times higher than that for the inhibition of thrombin generation (34 ng/ml, IC_50_ for tissue factor-induced thrombin generation) (33). Therefore, the plasma concentration of ART-123 could not reach the level required for directly inhibiting the thrombin activity in clinical settings (32, 33). Similar results were observed in the present study. A previous study reported that the highest plasma concentration at which no bleeding event was observed in the nonclinical toxicology studies in monkeys repeatedly administered ART-123 was 5,400 ng/ml (25). Maximum plasma ART-123 concentration measured in the present study was 3,291 ng/ml.

The present study also has some limitations. In critically ill patients, accurate estimation of the glomerular filtration rate is difficult in clinical settings (34). In particular, estimation of the glomerular filtration rate is considerably difficult in patients undergoing CRRT because it affects the serum creatinine and cystatin C levels. Although we measured the mean 24-h Ccr, the accuracy and precision of this method to estimate glomerular filtration rate have not been investigated. Furthermore, the urinary excretion rate of ART-123 was evaluated only during the first 24 h. Further studies to investigate the urinary excretion rate of ART-123 after 24 h may be necessary to clarify the reason for the low excretion rate of ART-123 (less than 20 % of the administered ART-123 even in patients with normal renal function) observed in this study. Lastly, we did not evaluate the pharmacodynamics of ART-123. Appropriate plasma concentration of ART-123 required to treat DIC was not clarified.

**What is known about this topic?**

- Recombinant human soluble thrombomodulin (ART-123) is a novel anticoagulant for disseminated intravascular coagulation (DIC) and widely used in clinical settings throughout Japan.
- In healthy subjects, ART-123 is excreted mainly via the kidneys.
- The pharmacokinetics of ART-123 in DIC patients with severe acute renal dysfunction has not yet been elucidated.

**What does this paper add?**

- ART-123 plasma concentrations were not different among patients with differing renal function.
- Renal dysfunction may not affect ART-123 plasma concentration after repeated administration.

### Conclusions

The plasma concentrations of ART-123 may not be different among patients with different renal function. Moreover, the pharmacokinetics of ART-123 was not different between patients with and without CRRT. ART-123 was well tolerated in these patients. The results of this study should be considered when deciding whether to maintain therapeutic plasma concentrations of ART-123 when treating DIC patients with acute renal dysfunction.

## Figures and Tables

**Table 1:** Patient characteristics.

Dose	380 U/kg				130 U/kg			
	Group 1	Group 2	Group 3	Group 4	Group 1	Group 2	Group 3	Group 4
	Ccr < 10.0	10.0 ≤ Ccr < 30.0	30.0 ≤ Ccr < 60.0	60.0 ≤ Ccr	Ccr < 10.0	10.0 ≤ Ccr < 30.0	30.0 ≤ Ccr < 60.0	60.0 ≤ Ccr
Number of patients	9	6	6	10	4	3	0	1
Ccr (ml/min), minimum – maximum (mean)	0.0–9.9 (5.6)	13.6–23.2 (18.1)	35.3–55.1 (47.9)	60.5–97.4 (78.4)	0.4–5.4 (3.3)	11.7–26.9 (20.5)	-	92.3–92.3 (92.3)
Age (years), mean ± SD	72.1 ± 10.3	63.7 ± 20.8	71.7 ± 13.3	72.6 ± 12.6	74.3 ± 8.7	78.7 ± 5.1	-	64.0
Sex, male/female	9/0	1/5	2/4	2/8	4/0	2/1	-	1/0
Weight (kg), mean ± SD	58.67 ± 7.50	55.02 ± 9.65	60.77 ± 19.92	51.85 ± 12.06	63.50 ± 12.06	72.13 ± 21.09	-	70.90
BMI (kg/m^2^), mean ± SD	21.44 ± 2.65	23.27 ± 5.29	24.73 ± 4.68	22.14 ± 4.36	23.99 ± 5.42	28.50 ± 7.91	-	25.12
APACHE II score, mean ± SD	21.6 ± 6.8	16.0 ± 7.3	22.3 ± 3.9	15.4 ± 4.5	24.5 ± 9.2	22.0 ± 9.6	-	8.0
DIC score, mean ± SD	5.3 ± 1.1	4.8 ± 1.6	5.8 ± 1.0	5.1 ± 1.3	5.8 ± 1.7	5.3 ± 2.3	-	4.0
SOFA score, mean ± SD	13.3 ± 2.5	9.7 ± 1.5	9.5 ± 2.2	7.7 ± 2.6	13.0 ± 3.6	11.3 ± 2.1	-	12.0
CRRT, Presence/absence	8/1	1/5	2/4	0/10	2/2	1/2	-	0/1
Underlying diseases that directly induced DIC, n								
Infectious disease	9	6	5	8	4	2	0	1
Respiratory	3	2	2	0	2	0	0	0
Abdominal organs	4	0	1	1	0	1	0	1
Urinary/ Reproductive organs	1	1	2	4	1	0	0	0
Soft tissue	0	2	0	3	0	0	0	0
Others	1	1	0	0	1	1	0	0
Non infectious	0	0	1	2	0	1	0	0

Ccr, 24-hour creatinine clearance on Day 1; BMI, body mass index; APACHE, Acute Physiology and Chronic Health Evaluation; DIC, disseminated intravascular coagulation; SOFA, Sequential Organ Failure Assessment; CRRT, continuous renal replacement therapy.

**Table 2:** Pharmacokinetic parameters of ART-123 during the first 24 hours.

Dose	380 U/kg				130 U/kg			
	Group 1	Group 2	Group 3	Group 4	Group 1	Group 2	Group 3	Group 4
	Ccr < 10.0	10.0 ≤ Ccr < 30.0	30.0 ≤ Ccr < 60.0	60.0 ≤ Ccr	Ccr < 10.0	10.0 ≤ Ccr < 30.0	30.0 ≤ Ccr < 60.0	60.0 ≤ Ccr
Number of patients	9	6	6	10	4	3	0	1
CL_tot_ (ml/hour/kg)	2.39 ± 0.988	2.76 ± 0.731	2.29 ± 0.515	3.56 ± 0.697	2.40 ± 0.720	2.16 ± 0.605	-	4.85
Vd (ml/kg)	82.7 ± 15.3	68.2 ± 24.3	71.4 ± 12.2	80.8 ± 16.8	75.0± 7.20	59.4± 8.04	-	97.7
C_max_ (ng/ml)	828 ± 156	1,110 ± 460	987 ± 294	807 ± 160	287 ± 41.6	390 ± 95.0	-	221
AUC_0–24_ (ng·hour/ml)	12,900 ± 2,910	14,400 ± 4,480	14,300 ± 3,010	11,100 ± 1,700	4,580 ± 847	5,580 ± 1,000	-	2,910
t_1/2_ (hours)	27.3 ± 12.0	16.8 ± 2.03	22.0 ± 2.93	16.2 ± 3.52	23.0 ± 5.72	19.9 ± 4.25	-	14.0
fe_0–24_ (%)	1.03 ± 0.895	4.58 ± 3.29	6.90 ± 3.41*	13.7 ± 4.77	1.04 ± 0.651	5.38 ± 1.29	-	18.2

Ccr, 24-hour creatinine clearance; CL_tot_, total body clearance; Vd, volume of distribution; C_max_, maximum plasma concentration; AUC_0–24_, area under plasma concentration-time curve from time 0 to 24 hours post dose; t_1/2_, elimination half-life; fe_0–24_, fractional excretion in urine from time 0 to 24 hours after dosing (Day 1 urine excretion rate); Data are represented as mean ± SD.

* fe_0–24_ was calculated using data for 5 patients, one patient was excluded from the analysis of fe_0–24_ owing to a urine collection error.
